# Nutritional Interventions for Patients with Mitochondrial POLG-Related Diseases: A Systematic Review on Efficacy and Safety

**DOI:** 10.3390/ijms231810658

**Published:** 2022-09-13

**Authors:** Zandra Overgaard Pedersen, Sonja Holm-Yildiz, Tina Dysgaard

**Affiliations:** Neuromuscular Research Unit, Department of Neurology, Rigshospitalet, 2100 Copenhagen, Denmark

**Keywords:** POLG, ketogenic diet, diet treatment, drug resistant epilepsy, diet therapy, carbohydrate-restricted diet, drug resistant epilepsy/diet therapy

## Abstract

Ketogenic diet is recommended as a treatment to reduce seizure frequency in patients with intractable epilepsy. The evidence and safety results are sparse for diet interventions in patients with pathogenic polymerase gamma (POLG) variants and intractable epilepsy. The aim of this systematic review is to summarize the efficacy of diet treatment on seizure frequency, clinical symptoms, and potential deleterious effect of liver involvement in patients with mitochondrial diseases caused by pathogenic POLG variants. Literature was searched in PubMed, Embase; and Cochrane in April 2022; no filter restrictions were imposed. The reference lists of retrieved studies were checked for additional literature. Eligibility criteria included verified pathogenic POLG variant and diet treatment. Overall, 880 studies were identified, providing eight case-reports representing nine patients eligible for inclusion. In eight of nine cases, clinical symptoms were improved; six out of nine cases reported improvements in seizure frequency. However, increasing levels of liver enzymes after initiating ketogenic diet were found in four of the nine cases, with one case revealing decreased levels of liver enzymes after initiating long-chain triglyceride restriction. Viewed together, the studies imply that ketogenic diet can have a positive impact on seizure frequency, but may induce progression of liver impairment in patients with pathogenic POLG variants.

## 1. Introduction

Mitochondria are small organelles found in nearly all cells, except red blood cells, and are responsible for production of adenosine triphosphate (ATP). ATP is the primary energy source for the maintenance of cell function [1]. Mitochondria are dynamic organelles in which the amount and size changes with the energy requirements of the cell [1]. Mitochondria contain their own DNA, mitochondrial DNA (mtDNA), that encodes for subunits of the mitochondria. Correct replication and repair of the mtDNA is essential to ensure ATP production in the mitochondrial respiratory chain. One important enzyme for correct replication and repair of mtDNA is polymerase *y*, which consists of a catalytic subunit (POLG) and two subunits (POLG2) [2,3]. More than 180 POLG variants have been identified, and found to cause a wide range of mitochondrial diseases (MD) [4]. Pathogenic variants or POLG or decreased activity of the enzyme are associated with mtDNA depletions and deletions [5,6], resulting in impaired ATP production and structural damage to the cell due to accumulation of abnormal mtDNA. The combination of depletion and deletion mediates a progressive disease course with high morbidity and mortality after disease onset [7,8,9]. An observational study found that median survival time from disease onset to death was 19 months for patients with pathogenic POLG variants [10]. The major cause of death was liver failure (32%), with a higher mortality rate in patients with liver impairment compared to those without [10]. Epilepsy was reported in 69% of the patients, and survival analysis showed lower survival rates with the presence of epilepsy [10].

Due to the wide distribution of mitochondria, mutations in POLG can affect many organs, and patients present with different phenotypes. The most common POLG-related diseases are Alpers–Huttenlocher syndrome (AHS) [11], myocerebrohepatopathy spectrum (MCHS), ataxia neuropathy spectrum (ANS) [12], myoclonus epilepsy myopathy with sensory ataxia (MEMSA), autosomal recessive progressive external ophthalmoplegia (arPEO) [13] and autosomal dominant progressive external ophthalmoplegia (adPEO) [5].

There is no treatment for diseases caused by pathogenic POLG variants. Treatment is therefore limited to symptomatic treatment, with an ineffective response to antiepileptic drugs (AEDS) [14,15]. Due to the impaired survival rate with the presence of epilepsy, alternative treatment strategies have been presented including ketogenic diet (KD). KD contains a high amount of fat and a reduced content of carbohydrates and traditionally aims for ketosis. KD has shown the ability to reduce the frequency of epileptic seizures [16] and is recommended in patients with refractory epilepsy when seizure freedom has not been obtained with two AEDs [17,18].

Several physiological mechanisms are linked to the effect of ketogenic diet in epileptic activity, Including an increased level of ketones and butanoic acid, can have an impact on neurons, by affecting the neurotransmitter balance, reduce inflammation and decrease cytokines as TNF-a and IL-6 levels [19]. Mechanism as metabolic shift from glucose metabolism to pentose phosphate pathway, decreasing of the intracellular reactive oxygen species (ROS), and an increasing in the mitochondrial oxidative defence by ketosis, through activation of k_ATP_-channels, whereby the activity of neurons decreases and reduces the frequency of seizures, assumes to be potential factors that could mediate reduction of seizures [20,21].

Even though ketogenic diet is recommended for patients with refractory epilepsy, there is no consensus on whether diet intervention should be enforced in patients with epilepsy due to POLG mutations. Therefore, the aim of this systematic review is to evaluate the physiological effect of nutritional interventions in patients with POLG mutation, considering seizure activity, liver affection, and clinical symptoms.

## 2. Results

### 2.1. Study Selection

**Figure 1:**
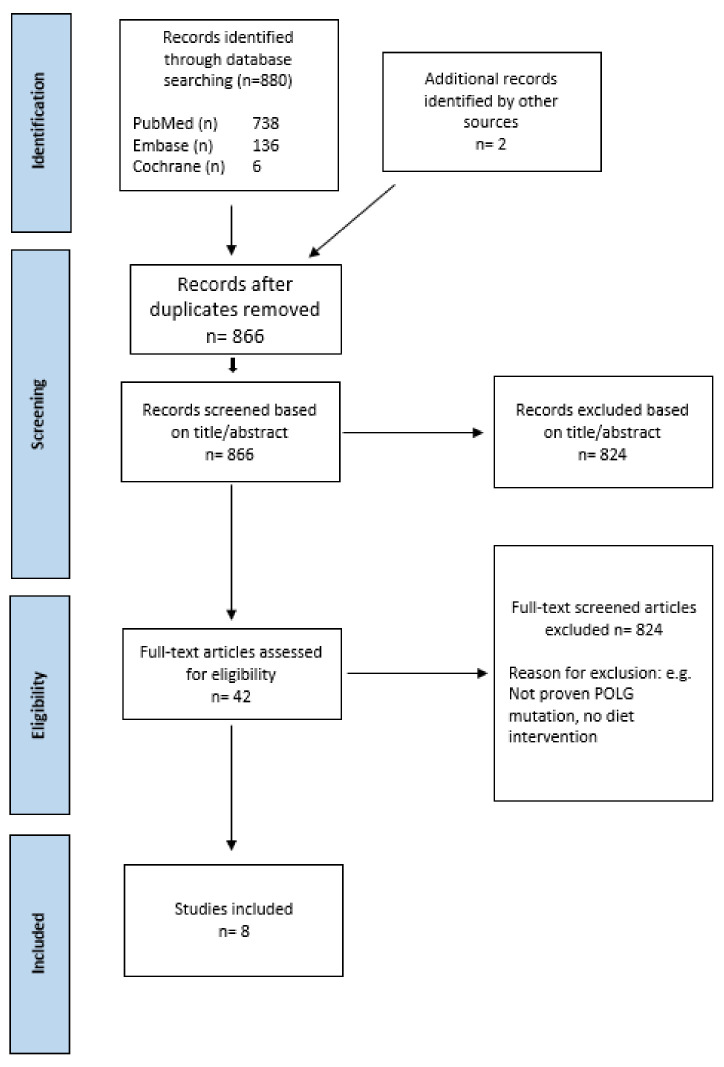
PRISMA flowchart.

The search strategy yielded 880 results, as presented in the PRISMA flowchart [22], Figure 1. For full reading, 42 papers were selected. Reasons for exclusion are summarized in Appendix B. Eight publications, describing nine cases, were included for detailed analysis [23,24,25,26,27,28,29,30]. Interrater reliability for full-text screening had Cohen’s kappa value of 1, indicating almost perfect agreement.

### 2.2. Study Characteristics

Study characteristics and diet specifications are presented in Table 1 and Table 2. All the studies included were case reports, published from 2009–2021. The tables present eight studies consisting of nine cases, of which 55% of the cases were females, 89% of the cases used KD as dietary treatment 89% of the cases were diagnosed with Alpers–Huttenlocher syndrome (AHS) or Alpers syndrome (AS).

### 2.3. Study Quality

In terms of case-report design, the general quality was low (category 4 [31]). As presented in Table 3, many of the studies were considered to have incomplete information according to CARE guidelines [32]. Categories including follow-up and outcomes, therapeutic intervention, and patient perspective were scored particularly low.

### 2.4. Primary Outcomes

#### 2.4.1. Epileptic Seizures

As presented in Table 4 improvements in seizure frequency due to nutritional interventions were reported in six of nine patients with pathogenic POLG variants [25,26,27,28,29,30]. Three studies did not find improvements in seizure frequency [23,24,27]. One study ceased KD after 2 weeks, due to lack of effect [27]. One study reported that the case experienced a relapse of seizures when ketones could no longer be measured. KD was used in eight of the nine studies, and increased seizure control was achieved in six of these. Plasma ketones were monitored in only three of the eight case studies.

#### 2.4.2. Liver Impairment

As presented in Table 5 accurate liver enzymes prior to and after diet intervention were specified in only two of the nine studies [24,26]. Four studies reported elevated liver enzymes post diet intervention [23,26,27]. In one study, liver enzymes were not reported but the authors found diffuse steatosis and cholestasis after autopsy [25]. The authors interpreted that these postmortem findings were compatible with severe liver impairment, and hypothesized that KD can potentially progress liver failure in patients with pathogenic POLG variants. One study observed decreasing liver enzymes after initiating diet intervention, with long-chain-fatty acid (LCT) restriction, while liver biopsy revealed cirrhosis, steatosis and mtDNA depletions [24].

#### 2.4.3. Clinical Symptom Improvements

As presented in Table 4, Eight of nine studies reported overall improvements in clinical symptoms including improved memory, return of bladder and bowel control, improvements in the ability to speak in sentences [26], improvements in aphasia [29], and improved response to AEDs [23,30].

### 2.5. Secondary Outcomes

#### 2.5.1. Compliance

Out of nine studies, none reported compliance to the diet intervention through diet registration, diet diary, or registration of enteral nutrition by bolus administrations (Table 2). Three of nine studies reported BHB values under the administration of KD, indicating compliance to the diet intervention [23,28].

#### 2.5.2. Macronutrient Composition and Effect

Eight of nine studies used KD as diet treatment, while one study used restriction of LCT as diet treatment [28] (Table 2). Positive improvements in clinical symptoms with diet intervention were found in eight of the nine study cases (Table 2).

#### 2.5.3. Nutritional Supplements

Nutritional supplements such as carnitine, riboflavin, Q10, and thiamine, were administrated in two of the nine studies [23,26] (Table 2).

#### 2.5.4. Effect on AEDs

One in nine studies reported reduction in AED consumption [23] (Table 4).

One study case reported improved response to AEDs after KD intervention [30] (Table 4).

## 3. Discussion

The aim of this systematic review was to evaluate the physiological effect on seizure activity, liver impairmentand clinical symptoms of nutritional interventions in patients with POLG mutations. KD may have a positive impact on seizure activity and development of status epilepticus, but some studies have suggested that KD can have a deleterious effect on liver function. Despite the overall low strength of our study, the data point toward a positive effect of KD on seizure activity, while the potential negative effect on liver affliction in patients with pathogenic POLG variant is unknown.

### 3.1. Seizures

One interventional study reported significant reduction of seizure frequency at β-hydroxybutyrate (BHB) > 4 mmol/L compared to BHB levels < 4 mmol/L [1]. When BHB levels reach >4 mmol/L the central nervous system initiates the use of ketones as the source for energy [2,3]. Ketones are more energy efficient due to changes in the mitochondrial energy production [3,4]. The effective seizure control at BHB levels > 4 mmol/L, indicate that the possible mechanisms such as reduction of ROS, decreasing inflammatory cytokines, changes in GABA/glutamate balance and an increased energy production is superior at BHB > 4 mmol/L. Only one of the included studies obtained ketone levels > 4 mmol/L in the presented case [5]. This case achieved complete cessation of seizures and noticeable improvements in EEG, despite the fact that the patient had a rapidly accelerated course of disease with death early after diagnosis [5]. In one study the seizures returned when it was no longer possible to measure ketones in the urine of the case [6] which underscores the importance of the presence of ketones in the treatment of epilepsies and indicate that the study cases where seizure control was not obtained, potentially could be a result of low BHB levels.

The recommendation is that to evaluate a potential effect of KD on seizure activity, the diet should be maintained for at least 3 months before it is appraised as ineffective [17]. In two studies, treatment with KD did not reveal any effect on seizure control [23,27]. In one of the studies, KD was ceased after 2 weeks of treatment, justified by the absence of effect [27]. The absence of effect in seizure control could potentially be attributed to an insufficient intervention period, and insufficient BHB levels which were not specified. The second study presented a 16-year-old female with rapidly advancing disease [23]; the absence of effect might be explained partly by the hormonal status of this case. This is based on an observational retrospective cohort study of 155 patients, which aimed to investigate the impact of gender, puberty, and pregnancy in patients with a confirmed pathogenic POLG variant [33]. Data revealed a trend toward gender differences, with seizures and onset of seizures appearing more common after onset of puberty in females compared to males [33]. Estrogen peaks with puberty, and is held to be pro-convulsant [33,34,35].

It remains unknown whether adaption to the KD in the two studies mentioned would have led to seizure control if the intervention had been continued longer, or if the treatment could have been initiated at a time with a different hormonal status.

Future studies should monitor BHB levels to evaluate the effect of KD, and make adjustments to the macronutrient composition in cases of insufficient or decreasing BHB levels.

### 3.2. Liver Impairment

In AHS, liver impairment and acceleration to severe liver failure are part of the disease pathogenesis due to the dysfunction of POLG [4]. Since KD can potentially induce steatosis and can elevate liver enzymes, it is unknown whether the inevitable steatosis and ultimate cirrhosis and liver failure is accelerated by KD in patients with AHS. Four studies reported elevated liver enzymes following KD treatment [23,26,27]. Thus, a potentially harmful effect mediated by KD on the liver can be neither confirmed nor rejected.

Interestingly, the only study that applied LCT-restriction instead of KD, measured decreasing liver enzymes, and presented the longest life-time duration of 56.5 months after onset of symptoms [24]. In that particular study, liver biopsy revealed cirrhosis, steatosis, and mtDNA depletions [24], indicating that liver affliction due to AHS was not prevented with LCT-restriction. However, the lowering of liver enzymes implies that seizure control can be improved along with reduction of further liver disease [6].

The number of studies investigating KD´s effect on liver function is sparse, and the information about natural history liver enzymes is limited. Thus, it is difficult to conclude to what extent KD had an adverse effect on liver function, or if liver affliction found in the presented studies merely reflected natural changes as a result of pathogenic POLG variants.

Future studies should assess liver enzymes, liver biopsies, and mtDNA deletions pre- and post diet treatment, to investigate to what extent liver impairment is caused by disease pathogenesis or diet treatment.

### 3.3. Mitochondrial Respiratory Chain (MRC)

An animal study in mice with complex I deficiencies showed that treatment with a fat-rich diet could reduce neurodegenerative symptoms and cerebral atrophy [36]. An additional mouse study has revealed that KD can promote complex II and IV activity, which theoretically can increase energy supply to neurons and promote neuronal survival [37]. Impaired complex I and IV activity has been reported in studies of patients with AHS due to pathogenic POLG variant [2,6,38]. In the studies considered here, only three of the eight presented MRC activity. The recommendations appraises that KD is indicated and beneficial in MD with complex I deficiencies and the utility in seizure frequency can be expected to be reduced with >70% [17]. This implies that systematic measurements of MRC in patients with pathogenic POLG variants should be mandatory, since patients with complex I or IV deficiency potentially could be prone to seizure control as an effect of KD intervention.

### 3.4. Macronutrient Composition and Effect

Carbohydrate restriction has revealed 15–30% reduction in seizure frequency in mice with epilepsy [39], and 50% reduction in seizure frequency in humans [40,41]. The reduced seizure frequency was shown to correlate with decreasing plasma glucose levels, while no association of BHB levels and seizure frequency was found [41]. RCT studies have revealed an association between decreased plasma insulin levels and memory improvements in patients with mild cognitive impairments [42]. In one of the presented studies, a low glycemic index treatment (LGIT) was used as a dietary treatment [29]. In this case, seizures ceased completely after initiating LGIT [29]. LGIT aims to stabilize and lower blood glucose (BG), instead of increasing ketone levels in plasma. Another of the presented studies reported improved memory after initiating a classic ketogenic diet (CKD) [26]. In this form of diet, carbohydrate intake is restricted to a maximum of three percent energy (E%) of total daily calories. The remaining 97 E% is contributed by fat and proteins. Plasma insulin levels and BG assumes to be stable and low, due to restricted carbohydrate intake, and minimal stimulation of the insulin secretion.

These studies indicate that stable and low BG may be an important parameter in reducing seizure frequency and that low plasma insulin levels may even be able to improve memory and should be addressed in future studies.

### 3.5. Compliance

The efficacy of KD depends on compliance to the restrictive diet therapy. One meta-analysis found that CKD was the most efficient diet intervention to reduce seizure frequencies, but that compliance was higher in Atkins [43]. Another study showed significant improved compliance to the Atkins ketogenic diet compared with CKD, in patients with refractory epilepsy [44]. Guidelines therefore recommend that patients should freely choose between diets e.g., medium-chain triglyceride ketogenic therapy (MCT), modified Atkins diet (MAD), LGIT, CKD, or KD, to ensure compliance [17]. See Appendix C for diet specifications.

None of the presented studies provided information about compliance to the diet treatment, and only a few measured ketones, so overall it is unknown to what extent the included patients were actually in ketosis or adhered to the diet intervention.

These findings emphasize that when introducing diet treatment, compliance should be monitored closely, to ensure that data concerning seizure activity, symptoms, and liver involvement reflects compliance to diet intervention. Moreover, this is perhaps even more important in patients with pathogenic POLG variants, since patients often have insufficient nutritional intake [9,10] which additionally complicates maintenance of KD.

### 3.6. AEDs

Data on pharmacodynamic interactions between AEDS and KD in humans are sparse and uncertain [17]. Ketones have been shown to increase the seizure-reducing activity of phenobarbital and carbamazepine in mice [45]. Ketosis could therefore potentially result in increased seizure control due to this interaction, and mediate a reduction of AED use. Only two of the eight studies reported the amount of AED use before and after diet intervention. One of these studies reported reduction in AED use after initiating a ketogenic diet [23] while another stated that AED treatment was not efficient until the introduction of KD [30]. Due to interactions between different AEDs and side effects, one of the aims in the treatment of epilepsy is to lower the amount of AED. When initiating KD, the efficacy of AEDs should be monitored, in order to reduce the use if applicable.

### 3.7. Nutritional Supplements

Thiamine deficiencies often occur with the presence of malnutrition. Thiamine is an essential co-enzyme in the glucose metabolism. Without the B1 vitamin, glucose is metabolized through anaerobic pathways and produces lactic acids. If thiamine deficiency occurs simultaneously with diet treatment it may cause adverse effects including lactic acidosis, Wernickes encephalopathy, and ultimately increased risk of mortality [46]. Information on supplementation has been provided in only two studies [23,26], while supplementation with thiamine was used in one study [23]. Nutritional screening could therefore be a valuable systematic procedure in patients with pathogenic POLG variants, to identify whether supplementation with thiamine is necessary.

## 4. Methods

This systematic review was conducted according to PRISMA (preferred reporting items for systematic reviews and meta-analyses) guidelines [22]. A detailed protocol was conducted prior to this systematic review, and is published on PROSPERO [CRD42022335722].

### 4.1. Search Strategy

Three databases were searched for literature; PubMed, Embase, and Cochrane. A pre-specified literature search was conducted in April 2022, without any search limitations. A librarian checked the search strategy. Reference lists were reviewed for additional literature. The search strategy is presented in Appendix A.

### 4.2. Study Selection

One author (Z.O.P.) screened and selected the papers according to titles and abstracts. Two authors (Z.O.P., S.H.-Y.) independently reviewed full-text articles and selected them according to exclusion criteria. Disagreements were solved by discussion with a third reviewer (T.D.).

### 4.3. Eligibility Criteria

Verified pathogenic POLG variant, diet intervention, and English language.

### 4.4. Exclusion Criteria

(i) Cases without proven pathogenic POLG variant; (ii) cases without data on effect of clinical outcomes post diet treatment; (iii) cases without nutritional intervention; (iv) cases only using nutritional supplements; (v) Animal studies.

Diet intervention was defined as any macronutrient manipulation.

### 4.5. Data Extraction

Excel spreadsheets designed specifically for this study were used for data management. One author extracted the data (Z.O.P.). Two authors (T.D., S.H-Y.) independently checked the data extraction to ensure accuracy and completeness. The following data were extracted: Author, publication year, gene mutation, gender, symptoms, age at onset of symptoms, medications, diagnosis, age at diagnosis, reason for death, age at death, diet intervention, age at onset of diet intervention, seizure frequency, EEG, development delays, efficacy of diet intervention on clinical outcomes, blood sample values.

### 4.6. Quality Appraisal and Interrater Reliability

Consensus-based clinical case reporting development, CARE guidelines [32,47] were applied to assess the quality of the included cases by rating each step with yes, no, or insufficient. Oxford Centre for Evidence-Based Medicine Levels of Evidence [48] were used to evaluate the overall quality of the included studies. Interrater reliability was calculated using Cohens kappa coefficient [49] for the selection of full text articles according to exclusion criteria between the two authors (Z.O.P., S.H-Y.).

### 4.7. Outcome Measures

The primary outcomes were the effect of diet intervention on epileptic seizures, liver impairment, and clinical symptom improvements. Secondary outcomes were compliance to diet intervention, measured by diet registration or beta-hydroxybutyrate, macronutrient composition and effect, nutritional supplements and reduction in AEDs or increased effect of AEDs after initiating diet intervention.

## 5. Conclusions

Treatment with ketogenic diet can have a positive impact on seizure frequency and clinical symptoms in patients with pathogenic POLG variant and intractable epilepsy. Adverse effects may include progressive liver affliction in patients with liver involvement due to a pathogenic POLG variant. Results of this systematic review should be interpreted with caution, as the results are based on case reports. Future studies should address the significance of ketogenic diet and its effect on liver impairment in patients with pathogenic POLG variant, considering the significance of stable blood glucose in terms of seizure frequency, monitor beta-hydroxybutyrate levels, and investigate compliance to the diet treatment by maintaining dietary records.

## 6. Limitations

Several limitations are associated with the current study. The included literature was exclusively based on case reports, and several limitations were identified according to CARE guidelines. Moreover, dissimilar competing gene mutations in each individual patient can impact disease presentation and affect the efficacy of ketogenic diet on an exclusively pathogenic POLG variant. Missing information on compliance and the absence of ketosis measurements and ketone levels made it difficult to evaluate whether patients achieved the full potential of the dietary treatment. A further significant limitation was that only patients with pathogenic POLG variants diagnosed with Alpers–Huttenlocher syndrome or Alpers syndrome were included, where liver impairment is part of the disease pathogeneses. It is therefore impossible to deduce from the presented cases a conclusion about the impact of ketogenic diet on other diseases caused by pathogenic POLG variants.

## Figures and Tables

**Table 1:** Study characteristics: Column (2) indicates the genetic pathogenic variant; (3) lists deficiencies or reduced activity of the mitochondrial respiratory chain complexes; (4) gender; (5–10) contain disease-specific information; (11–12) contains information about medication.

Authors	POLG Mutation	Complex Deficiencies	Gender	Symptoms at Onset	Age at Onset of Symptoms	Diagnosis	Age at Diagnosis	Age at Death	Reason for Death	Co-Medication	Valproate
Scalais et al., 2011 [24]	C.1399G>A + C.2542G>A	Complex IV	F	Hypoglycemia, hyperlactatemia	3–5 mo.	AS	3–5 y	5 y	Respiratory failure	AED	Avoided
Koessler et al., 2021 [23]	C.1399G>A	N/A	F	Refractory status epilepticus	16 y	AHS	16 y	16 y	Apnea	LEV+PB+LCM	2 days
Khan et al., 2011 [25]	C.1399>A + C.3562T>C	Complex IV+V	M	Epileptic seizures	9 mo.	AHS	9 mo.	14 mo.	Heart failure, Respiratory failure	N/A	N/A
Joshi et al., 2009 [26]	C.2243G>C + C.2480+1g>A	Normal	F	Status epilepticus	31 mo.	AHS	55 mo.	66 mo.	Respiratory failure	LEV+NP+ESM	Avoided
Spiegler et al., 2011 [27]	C.911 T>G + C3434insGAGG	N/A	M	Developmental delay	15 mo.	AHS	33 mo.	35 mo.	N/A	N/A	Short period
Spiegler et al., 2011 [27]	C.1399G>A + C.844T>G	N/A	F	Developmental delay	18 mo.	AHS	45 mo.	46 mo.	N/A *	LCM+TPM	Short period
O’Connor et al., 2014 [28]	N/A **	N/A	M	Status epilepticus	10 mo.	AHS	N/A	N/A ****	Liver failure	N/A ***	N/A
Martikainen et al., 2012 [29]	C.2243G>C	N/A	M	Seizures, headaches, visual and speech disturbances	26 y	N/A	N/A	Alive in the process	Alive in the process	PHT+OXC+LEV	N/A
Cardenas and Amato, 2010 [30]	C.911T>G + C.1174C>G + 3240–3242 duplication	N/A	F	Epilepsia partialias continua	14 mo.	AHS	14 mo.	19 mo.	Liver failure, status epilepticus	Multidrug	Avoided

N/A = Not available, F = female, M = male, Mo = months, AS= Alpers syndrome, AHS = Alpers–Huttenlocher syndrome, LEV = levitiracetam, PB = phenobarbital, LCM = lacosamide, NP = nitrazepam, ESM = ethosuximid, TPM = topiramate, PHT = phenytoin, OXC = oxcarbazepine. * Authors suppose bowel obstruction. ** Verified POLG mutation. *** Resistant for seven AEDs. **** Authors informed that death occured soon after diagnosis.

**Table 2:** Diet specifications: Details of the diet treatment, ketone levels, diet registration, age at initiating diet intervention, duration of diet treatment, and use of nutritional supplements.

Authors	Diet Treatment	Beta-Hydroxybutyrate	Diet Registration	Age at Initiating Diet Treatment	Duration of Diet Treatment	Nutritional Supplements
Scalais et al., 2011 [24]	LCT-restrictions *	N/A	N/A	5.5 mo. (63 days after onset)	N/A	N/A
Koessler et al., 2021 [23]	4:1 KD	>2 mmol/L day 5 on KD	N/A	16 y (9 days after onset)	3 mo.	riboflavin, Q10, thiamine
Khan et al., 2011 [25]	KD	N/A	N/A	13 mo.	N/A	N/A
Joshi et al., 2009 [26]	CKD	N/A	N/A	55 mo.	Until death (66 mo.)	Carnitine
Spiegler et al., 2011 [27]	KD (PEG)	N/A	N/A	33 mo.	Discontinued after 2 weeks	N/A
Spiegler et al., 2011 [27]	KD (PEG: Ketocal advanced)	N/A	N/A	44 mo.	Until death (46 mo.)	N/A
O´Connor et al., 2014 [28]	4:1 KD	5.1 mmol/L	N/A	10.5 mo. (15 days after onset)	Until death	N/A
Martikainen et al., 2012 [29]	LGIT	N/A	N/A	26 y (7 days after onset)	Continued	N/A
Cardenas and Amato, 2010 [30]	KD (NG)	Measured **	N/A	14 mo.	N/A	N/A

**Abbreviations:** LCT = Long-chain triglycerides, NG = nasogastric tube, PEG = percutaneous endoscopic gastrostomy, CKD = classic ketogenic diet, KD = ketogenic diet, LGIT = low glycemic index treatment, mo. = months, y = years, N/A = not available. * NG supplementary fractional tube feeding + IV glucose + NG corn starch + LCT or MCT supplements. ** Measured precise levels not specified; unable to make ketones in urine after discharge.

**Table 3:** Quality assessment according to CARE guidelines.

	Scalais et al., 2011 [24]	Koessler et al., 2021 [23]	Khan et al., 2011 [25]	Joshi et al., 2009 [26]	Spiegler et al., 2011 [27]	Spiegler et al., 2011 [27]	O´Connor et al., 2014 [28]	Martikainen et al., 2012 [29]	Cardenas and Amato, 2010 [30]
Title	I	I	I	N	N	N	N	N	I
Keywords	I	Y	Y	N	I	I	I	I	N
Abstract	Y	Y	Y	Y	I	I	Y	Y	Y
Introduction	Y	Y	Y	Y	Y	Y	Y	Y	N
Patient information	Y	Y	Y	Y	I	I	I	I	Y
Clinical findings	Y	I	Y	Y	Y	Y	I	I	I
Timeline	Y	Y	Y	Y	I	I	I	I	I
Diagnostic assessment	Y	Y	Y	Y	Y	Y	I	Y	Y
Therapeutic intervention	I	Y	I	Y	I	I	I	I	I
Follow-up and outcomes	I	Y	I	I	I	I	I	I	I
Discussion	Y	Y	Y	Y	Y	Y	I	Y	I
Patient perspective	N	N	N	Y	N	N	N	I	N
Informed consent	I	I	Y	I	I	I	I	I	I

Y = yes, N = no, I = insufficient.

**Table 4:** Efficacy of diet treatment measured by reduction in frequency of seizures, change in pathogenic EEG, clinical symptom improvements, and reduction in antiepileptic medication (AED).

Authors	Reduction in Epileptic Seizures	EEG Improvements	Clinical Symptom Improvements	Effect on AEDs
Scalais et al., 2011 [24]	%	%	X ******	N/A
Koessler et al., 2021 [23]	%	X	X *****	X ************
Khan et al., 2011 [25]	X	%	X********	N/A
Joshi et al., 2009 [26]	X **	X	X *	N/A
Spiegler et al., 2011 [27]	% ***	%	%	N/A
Spiegler et al., 2011 [27]	X	%	X *********	N/A
O´Connor et al., 2014 [28]	X	X	X **********	N/A
Martikainen et al., 2012 [29]	X	%	X ****	N/A
Cardenas and Amato, 2010 [30]	X *******	%	X ***********	X ***********

N/A = Not available. * Increased alertness, improved memory, return of bladder and bowel control, ability to walk with assistance, speak in three- or four-word sentences, left-hand twitching ceased. ** Total cessation of seizures in 7 months. *** No effect of KD. Diet treatment ceased after 2 weeks. **** Headaches and aphasia reduced, and visual fields normalized. ***** Improvement of symptoms and stabilisation of general condition over a short time. ****** Improvements of abdominal distension and jaundice. Liver enzymes normalized. ******* Relapse in seizures, when the case was unable to make ketones in the urine. ******** Total cessation of seizures. ********* Increased alertness and cessation of seizures. ********** Reduction in seizure frequency and improvements in pathogenic EEG. *********** Reduction in seizure frequency and increased respond to AEDs. ************ Reduction in AED use.

**Table 5:** The Impact of Diet Treatment on Liver Samples Measured by ASAT, ALAT and GGT.

Authors	ASAT Pre Diet Treatment	ASAT Post Diet Treatment	ALAT Pre Diet Treatment	ALAT Post Diet Treatment	GGT Pre Diet Treatment	GGT Post Diet Treatment
Scalais et al., 2011 [24]	244 Ul/l	100 Ul/L ****	273 Ul/l	47 Ul/l ****	N/A	N/A
Koessler et al., 2021 [23]	N/A	704 U/l *	N/A	782 U/l *	N/A	1700 U/l
Khan et al., 2011 [25]	Moderately elevated **	N/A ***	Moderately elevated **	N/A	Moderately elevated	N/A ***
Joshi et al., 2009 [26]	51–73 U/L	94–112 U/L	30–47 U/L	60–101 U/L	N/A	N/A
Spiegler et al., 2011 [27]	Moderately elevated **	81 U/L *****	Moderately elevated **	61 U/L *****	N/A	N/A
Spiegler et al., 2011 [27]	Moderately elevated **	163 U/L *****	Moderately elevated **	130 U/L *****	N/A	N/A
O´Connor et al., 2014 [28]	N/A	N/A	N/A	N/A	N/A	N/A
Martikainen et al., 2012 [29]	N/A	N/A	N/A	N/A	N/A	N/A
Cardenas and Amato, 2010 [30]	N/A	N/A	N/A	N/A	N/A	N/A

ASAT = aspartate amino transferase, ALAT = alanine amino transferase, GGT = gammaglutamyl transferase. * Normalized within 4 days. ** Mild elevations. Not specified further. *** Autopsy showed diffuse steatosis and fatty accumulation. **** Liver impairment reversed with LCT-restriction. Liver biopsy revealed cirrhosis, steatosis, and mtDNA depletionens in the liver. ***** Highest measured liver enzymes.

## Data Availability

Not applicable.

## Appendix A

### Search Strategy

**Table A1:** PubMed.

Search	Query
#1	DNA Polymerase gamma [MESH]
#2	DNA-Directed DNA Polymerase/genetics [MESH]
#3	Mitochondrial Diseases/therapy [MESH]
#4	DNA-Directed DNA Polymerase [MESH]
#5	POLG protein: human [MESH]
#6	Status Epilepticus/pathology [MESH]
#7	POLG1 [Title/Abstract]
#8	POLG mutation [Title/Abstract]
#9	nDNA mutation [Title/Abstract]
#10	POLG [Title/Abstract]
#11	Alpers huttenlocher syndrom * [Title/Abstract]
#12	MEMSA [Title/Abstract]
#13	Myoclonic epilepsy myopathy sensory ataxia [Title/Abstract]
#14	SCAE [Title/Abstract]
#15	Spinocerebellar ataxia with epilepsy [Title/Abstract]
#16	ataxia neuropathy spectrum [Title/Abstract]
#17	MIRAS [Title/Abstract]
#18	SANDO [Title/Abstract]
#19	#1 OR #2 OR #3 OR #4 OR #5 OR #6 OR #7 OR #8 OR #9 OR #10 OR #11 OR #12 OR #13 OR #14 OR #15 OR #16 OR #17 OR #18 [Hits:51,680].
#20	Diet: Ketogenic * [MESH]
#21	Glycemic Index [MESH]
#22	Diet: Ketogenic/methods * [MESH]
#23	Diet: High-Fat [MESH]
#24	Diet * [MESH]
#25	Epilepsy/diet therapy [MESH]
#26	Ketogenic diet [Title/Abstract]
#27	low glycaemic index diet [Title/Abstract]
#28	High fat diet [Title/Abstract]
#29	Modified atkins diet [Title/Abstract]
#30	#20 OR #21 OR #22 OR #23 OR #24 OR #25 OR #26 OR #27 OR #28 OR #29 [Hits:1,343,284]. #19 AND #30 [Hits:738].

**Table A2:** Cochrane.

Search	Query
#1	DNA Polymerase gamma [MESH]
#2	DNA-Directed DNA Polymerase [MESH]
#3	POLG1 [Title:Abstract:Keyword]
#4	POLG mutation [Title:Abstract:Keyword]
#5	POLG [Title:Abstract:Keyword]
#6	Alpers huttenlocher syndrome [Title:Abstract:Keyword]
#7	MEMSA [Title:Abstract:Keyword]
#8	SCAE [Title:Abstract:Keyword]
#9	Spinocerebellar ataxia with epilepsy [Title:Abstract:Keyword]
#10	ataxia neuropathy spectrum [Title:Abstract:Keyword]
#11	MIRAS [Title:Abstract:Keyword]
#12	SANDO [Title:Abstract:Keyword]
#13	#1 OR #2 OR #3 OR #4 OR #5 OR #6 OR #7 OR #8 OR #9 OR #10 OR #11 OR #12 [Hits 486].
#14	Diet: Ketogenic [MESH]
#15	Diet theraphy [MESH]
#16	Glycemic Index [MESH]
#17	“ketogenic diet” [Title:Abstract:Keyword]
#18	“High fat diet” [Title:Abstract:Keyword]
#19	“Atkins diet” [Title:Abstract:Keyword]
#20	#14 OR #15 OR #16 OR #17 OR #18 OR #19 [Hits: 8234] #13 AND #20 [Hits 6]

**Table A3:** Embase.

Search	Query
#1	DNA directed DNA polymerase gamma [Map term]
#2	Alpers disease [Map term]
#3	Complex 1 deficiency [Map term]
#4	Mitochondrial encephalopathy [Map term]
#5	Multiple acyl coa dehydrogenase deficiency [Map term]
#6	POLG [Keyword]
#7	Alpers huttenlocher [Keyword]
#8	MEMSA [Keyword]
#9	SCAE [Keyword]
#10	Spinocerebellar ataxia with epilepsy [Keyword]
#11	MIRAS [Keyword]
#12	SANDO [Keyword]
#13	#1 OR #2 OR #3 OR #4 OR #5 OR #6 OR #7 OR #8 OR #9 OR #11 OR #12 [Hits: 5334].
#14	Low glycemic index diet [Map term]
#15	Low fat diet [Map term]
#16	Methionine/choline deficient diet [Map term]
#17	Ketogenic diet [Map term]
#18	High methionine diet [Map term]
#19	Low carbohydrate diet [Map term]
#20	High-protein low-carbohydrate diet [Map term]
#21	Lipid diet [ Map term]
#22	Atkins diet [Map term]
#23	Protein diet [Map term]
#24	Mediterranean diet [Map term]
#25	Ketogenic diet [Title]
#26	Low glycemic-index diet [Title]
#27	Low carbohydrate diet [Title]
#28	Modified Atkins diet [Title]
#29	#14 OR #15 OR #16 OR #17 OR #18 OR #19 OR #20 OR #21 OR #22 OR #23 OR #24 OR #25 OR#26 OR #27 OR #28 [Hits:89936]. #13 AND #29 [Hits:136].

## Appendix B

**Table A4:** Specification for reasons for exclusions of identified studies.

Study/Year	Reason for Exclusion
Hattori et al., 2001 [50]	MD not caused by POLG variant
Bachmann-Gagescu et al., 2009 [51]	MD not caused by POLG variant
Kumagi, 1999 [52]	MD not caused by POLG variant
Barzegar & Hashemilar, 2007 [53]	Missing information about diet intervention
Malojcic et al., 2004 [54]	Not clinical proven POLG mutation (LEIGH)
Seessle et al., 2012 [55]	Not clinical proven POLG mutation (MELAS)
Roe & Brunengraber, 2015 [56]	Not clinical proven POLG mutation
Daniel et al., 2015 [57]	No diet intervention
Di pisa et al., 2012 [58]	MD not caused by POLG variant
Pons et al., 2000 [59]	MD not caused by POLG variant
Gong et al., 2021 [60]	Not clinical proven POLG mutation (LEIGH)
Teitelbaum et al., 2002 [61]	Missing information about diet intervention
Morava et al., 2006 [62]	Not clinical proven POLG mutation
Beghin et al., 2016 [63]	Not clinical proven POLG mutation
Laugel et al., 2007 [64]	Not clinical proven POLG mutation (LEIGH NDUFV1 mutation)
Llingworth et al., 2012 [65]	Authors contacted for access
Zweers et al., 2020 [66]	Not clinical proven POLG mutation
Nangia et al., 2012 [67]	Not clinical proven POLG mutation
Kang et al., 2006 [68]	Not clinical proven POLG mutation
Craigen et al., 1996 [69]	Not clinical proven POLG mutation LEIGH (E3 deficiency Maple syrup urine disease)
Wijburg et al., 1992 [70]	Not clinical proven POLG mutation LEIGH (PDHC deficiency)
Ahola et al., 2016 [71]	MD not caused by POLG variant
Wexler et al., 1997 [72]	MD not caused by POLG variant (PDHC deficiency)
Barnerias et al., 2010 [73]	MD not caused by POLG variant
Suntum et al., 2017 [74]	MD not caused by POLG variant
Deberles et al., 2020 [75]	MD not caused by POLG variant
Kang et al., 2007 [76]	MD not caused by POLG variant
Theunissen et al., 2017 [77]	MD not caused by POLG variant
Berio, A 1994 [78]	Authors contacted for access
Kanabus et al., 2016 [79]	Other MD disease (LEIGH) Supplement intervention
Peterson, P. L, 1995 [80]	Not clinical proven POLG
Villarroya et al., 2019 [81]	Authors contacted for access
Foschi et al., 2015 [82]	MD not caused by POLG variant
Sato-shirai et al., 2021 [83]	MD not caused by POLG variant (LEIGH ECHS1D gene)

## Appendix C

**Table A5:** Specification for different ketogenic diet treatments.

	General Recommendation (Nordic Nutrition Recommendations)	4:1/CKD	MAD/Atkins	LGIT	MCT-KD
Fedt	25–40 E%	90 E%	70 E%	45 E%	70 E%
Protein	10–20 E%	7 E%	25 E%	28 E%	10 E%
Kulhydrater	45–60 E%	3 E%	5 E%	27 E%	20 E%

4:1/CKD = Classic ketogenic diet. MAD = modified Atkins diet. LGIT = Low glycemic-index treatment. MCT = Medium-chain triglyceride treatment.
